# Correction: Aspirin better than clopidogrel on major adverse cardiovascular events reduction after ischemic stroke: A retrospective nationwide cohort study

**DOI:** 10.1371/journal.pone.0232016

**Published:** 2020-04-15

**Authors:** Amelia Nur Vidyanti, Lung Chan, Cheng-Li Lin, Chih-Hsin Muo, Chung Y. Hsu, You-Chia Chen, Dean Wu, Chaur-Jong Hu

After publication of this article [1], the authors contacted *PLOS ONE* to clarify that they did not obtain prospective institutional review board approval to conduct their study. Upon editorial review and consultation with the Academic Editor, it was determined that the article meets the journal’s standards for ethical oversight and that approval by an ethics committee was not necessary given that all data analyzed in the study were fully anonymized prior to access and analysis by the authors.

In light of this update, the final two sentences of the second paragraph in the Study design and population subsection of the Materials and methods are incorrect. The correct sentence is: All data analyzed in the study were fully anonymized prior to access and analysis by the authors.
